# Response to Vaccine-Derived Polioviruses Detected through Environmental Surveillance, Guatemala, 2019

**DOI:** 10.3201/eid2908.230236

**Published:** 2023-08

**Authors:** Rodrigo Rodríguez, Elisa Juárez, Concepción F. Estívariz, Coralia Cajas, Gloria Rey-Benito, María Olga Bautista Amézquita, Stacey Jeffries Miles, Oscar Orantes, María Cecilia Freire, Ana-Elena Chévez, Leticia Castillo Signor, Leanna Sayyad, Claudia Jarquin, Emilia Cain, Andrea Patricia Villalobos Rodríguez, Linda Mendoza, Carlos A. Ovando, Haroldo de Jesús Barillas Mayorga, Ericka Gaitán, Antonio Paredes, Hanen Belgasmi-Allen, Lorena Gobern, Marc Rondy

**Affiliations:** Pan American Health Organization, Washington, DC, USA (R. Rodríguez, G. Rey-Benito, M.C. Freire, A.-E. Chévez,^,^ E. Cain, A.P. Villalobos Rodríguez);; Ministry of Public Health and Social Assistance, Guatemala City, Guatemala (E. Juárez, M.O. Bautista Amézquita, L. Castillo Signor, L. Mendoza, C.A. Ovando, H. de J. Barillas Mayorga, E. Gaitán, A. Paredes, L. Gobern);; US Centers for Disease Control and Prevention, Atlanta, Georgia, USA (C.F. Estívariz, S.J. Miles, L. Sayyad, H. Belgasmi-Allen);; Pan American Health Organization, Guatemala City (C. Cajas, O. Orantes, C. Jarquin, M. Rondy);; Poliovirus Regional Reference Laboratory, Malbrán Institute, Buenos Aires, Argentina (M.C. Freire);; National Health Laboratory, Guatemala City (L. Mendoza)

**Keywords:** poliomyelitis, viruses, polio, poliovirus, Guatemala, environmental monitoring, vaccination

## Abstract

Guatemala implemented wastewater-based poliovirus surveillance in 2018, and three genetically unrelated vaccine-derived polioviruses (VDPVs) were detected in 2019. The Ministry of Health (MoH) response included event investigation through institutional and community retrospective case searches for acute flaccid paralysis (AFP) during 2018–2020 and a bivalent oral polio/measles, mumps, and rubella vaccination campaign in September 2019. This response was reviewed by an international expert team in July 2021. During the campaign, 93% of children 6 months <7 years of age received a polio-containing vaccine dose. No AFP cases were detected in the community search; institutional retrospective searches found 37% of unreported AFP cases in 2018‒2020. No additional VDPV was isolated from wastewater. No evidence of circulating VDPV was found; the 3 isolated VDPVs were classified as ambiguous VDPVs by the international team of experts. These detections highlight risk for poliomyelitis reemergence in countries with low polio vaccine coverage.

Poliomyelitis is a highly infectious disease, caused by poliovirus serotypes 1, 2, and 3, that primarily affects children <5 years of age. The main risk factors for poliovirus transmission are low immunization coverage, poor sanitation, and high population density (*1*). Since the worldwide launch of the Global Polio Eradication Initiative (GPEI) in 1988, polio cases have declined by >99% (*2*). Strategies to reduce the number of polio cases globally have focused on achieving high polio vaccination coverage and implementing robust acute flaccid paralysis (AFP) surveillance (*3*).

Administration of the injectable inactivated poliovirus vaccine (IPV) or live attenuated oral poliovirus vaccine (OPV, Sabin-strain virus types) can prevent poliomyelitis. IPV induces humoral protection, whereas OPV induces humoral and mucosal immunity and limits viral shedding, reducing person-to-person transmission (*4*). However, in areas with low vaccination coverage and poor sanitation, using OPV may exceptionally result in the emergence of vaccine-derived polioviruses (VDPVs) (*3*,*5*,*6*). VDPVs are classified as cVDPV (circulating VDPV, when there is evidence of community transmission), iVDPV (immunodeficiency-associated VDPV, isolated from persons with primary immunodeficiencies), or aVDPV (ambiguous VDPV, isolated from persons without immunodeficiency or from wastewater samples without evidence of transmission). aVDPVs are generally considered to be of low public health significance; however, they can still be an indicator of low vaccination coverage and poor sanitation, which can create the conditions for the emergence and circulation of potentially more dangerous cVDPVs. In the past decade, cVDPV outbreaks have caused >2,700 poliomyelitis cases globally (*7*–*10*).

In Guatemala, the last case of clinical poliomyelitis was detected in 1990 (*11*); the country routinely vaccinates children against poliomyelitis with trivalent IPV and bivalent (serotypes 1 and 3) OPV (bOPV) (*12*). Because of low vaccination coverage (<90% with third dose of polio-containing vaccine in 2017) and poor AFP surveillance indicators, the Regional Certification Commission (RCC) classified Guatemala as a high-risk country for polio reemergence in 2018. To complement AFP surveillance, the Pan American Health Organization’s (PAHO) Technical Advisory Group recommended implementing wastewater-based environmental surveillance in high-risk settings in the Americas in 2016 (*13*). After that recommendation was published, the Guatemala Ministry of Health (MoH) implemented poliovirus environmental surveillance in 2 urban municipalities in Guatemala in November 2018 (*14*).

The detection of 3 VDPVs from wastewater sampled in January, March, and December 2019 led the Guatemala MoH to implement a series of activities to classify these events and minimize the risk for transmission in the population (Figure). In July 2021, a team of international experts evaluated that response, according to the Global Polio Eradication Initiative’s (GPEI) Poliovirus Outbreak Response Assessment (OBRA) guidelines (*15*). We present the results of the investigation conducted by the MoH and the OBRA evaluation of their response.

**Figure:**
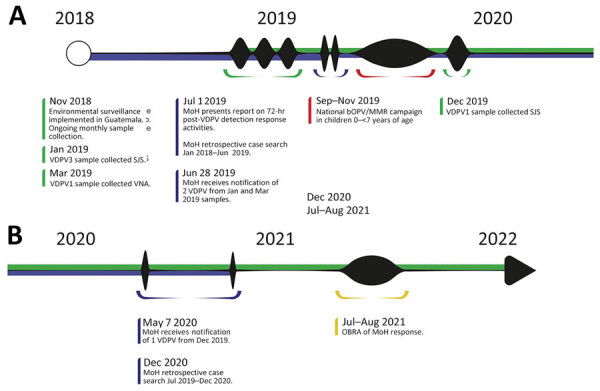
Timeline of MoH VDPV detection response activities and OBRA evaluation, Guatemala, 2018–2022.1 Green represents environmental surveillance-related activities. Blue represents MoH response activities. Red represents supplementary immunization campaign. Yellow represents OBRA. MoH, Guatemala Ministry of Health; OBRA, Poliovirus Outbreak Response Assessment; VDPV, vaccine-derived polioviruses.

## Methodology

### Environmental Surveillance, VDPV Detection, and Genomic Sequencing

Guatemala MoH implemented monthly collection of wastewater samples in November 2018. Three sampling sites in 2 municipalities were selected for logistical and financial reasons. The selected municipalities, Villa Nueva (VNA) and San Juan Sacatepéquez (SJS), were of high risk for poliovirus transmission (*16*): high population density (1,573 inhabitants/km^2^ in VNA and 943 inhabitants/km^2^ in SJS), OPV coverage <90% in 2014–2017, underperforming AFP surveillance indicators (<1 case/100,000 children <15 years of age), and poor sanitation conditions. In each municipality, the MoH revised wastewater networks to define 3 sampling points that would lead to a maximum population size unaffected by polluting industrial plants.

During November 2018–July 2021, trained MoH staff monthly collected a 1 L wastewater sample from each site and transported them in cold chain (2°C–8°C) the same day to the National Health Laboratory. Samples were stored at −80°C, then shipped at −20°C to the US Centers for Disease Control and Prevention (CDC) reference laboratory for polioviruses to be processed and tested for the presence of poliovirus (*16*–*18*). All poliovirus isolates underwent genomic sequencing and analysis of the region coding the viral protein 1 surface protein for nucleotide substitutions compared with the parent Sabin strains.

### Event Investigation

To classify the isolated VDPVs, the MoH relied on preexisting national protocols. Within 72 hours of receiving the notification of VDPV detection in wastewater samples, MoH personnel conducted health facility and community-based AFP case search in the areas surrounding the sampling sites. In each site, after obtaining verbal consent from parents, they also collected and tested stool samples for poliovirus from 20 healthy children <5 years of age without a history of OPV vaccination in the previous 30 days.

MoH epidemiologists retrospectively reviewed medical records from all 42 national hospitals for patients admitted during January 2018–December 2020 who had diagnoses compatible with polio or AFP, identified by codes from the International Classification of Diseases, 10th Revision (ICD-10). The retrospective record search was conducted in 2 phases during July 2019–December 2020. Finally, MoH epidemiologists revised hospital-based information systems to identify reported cases of primary immunodeficiency in 2018–2019 in patients residing in the affected municipalities.

### Routine AFP Surveillance

During 2017–2020, a total of 42 reporting hospitals conducted AFP surveillance in Guatemala. The surveillance system was classified as good quality if it reported >1 AFP case/100,000 children <15 years of age/year. Of reported AFP cases, >80% should have adequate sample collection and 80% should include an investigation conducted within 48 hours (*19*).

### Assessment of Polio Immunization Coverage and Supplementary Immunization Activities

Since 2016, Guatemala routinely vaccinates children with 1 IPV dose at 2 months of age, 2 bOPV doses at 4 and 6 months of age, and 2 bOPV booster doses at 18 months and 4 years of age (*12*). A nationwide catch-up vaccination campaign for bOPV and measles, mumps, rubella (MMR) was already planned for September–October 2019 for children 1–6 years of age. The detection of VDPVs led officials to lower the eligibility age to 6 months for bOPV and mobilize additional funding to support vaccination activities, a nationwide communication campaign, and technical support at the national and local level.

We calculated routine vaccination coverage during 2017–2020 using the number of third doses of bOPV registered in the national electronic immunization system (individual personal data) as numerator. This national system is exclusively used by MoH vaccination centers and may include social security institute centers on some occasions (based on personal willingness to share data). We calculated the bOPV vaccination campaign coverage using the number of doses registered in an electronic immunization system designed for the vaccination campaign (aggregated data). We estimated population denominators using national census-based projections.

### OBRA Implementation

In July 2021, an OBRA team virtually evaluated the MoH’s response to 3 VDPV detections in 4 key components: response planning and coordination, AFP surveillance sensitivity, vaccination, and health promotion and social mobilization. The team consisted of vaccination, epidemiologic surveillance, and laboratory experts from PAHO and CDC, as well as a national facilitator from the National Polio Elimination Certification Committee (NCC). The assessment involved reviewing technical documentation such as AFP and environmental surveillance protocols and vaccination reports; conducting interviews with national and local authorities, health personnel, and the National Polio Eradication Commission; and participating in work sessions with the OBRA team to discuss the data and provide recommendations. The assessment results were communicated to the MoH and local public health authorities through a virtual debriefing in August 2021 and a written report in September 2021.

### Ethics Considerations

The Guatemala MOH determined that the community survey, including collection of stool specimens, did not constitute human subjects research. We obtained verbal consent from household members who participated in the community survey and from parents of children <5 years of age who provided a specimen. The information obtained would be used to guide the public health response to a potential outbreak and did not involve risk to participants’ health.

## Results

### Environmental Surveillance, VDPV Detection, and Genomic Sequencing

During November 2018–July 2021, we collected 192 wastewater samples in SJS and VNA. We detected >1 enterovirus in 97% of samples collected in SJS and in 96% of samples collected in VNA. We isolated Sabin-like vaccine poliovirus in 83 samples (43%), Sabin type 1 poliovirus in 20% of samples and type 3 in 33%.

We detected 3 genetically unrelated VDPVs in 2019 (Appendix Figure). We identified a VDPV3 with 11 nt changes from Sabin type 3 in a January 2019 sample from Aldea Cruz Blanca, SJS, and a VDPV1 with 11 nt changes from Sabin type 1 in a March 2019 sample from Platanitos. We reported detecting those 2 VDPVs to the Guatemalan MoH on July 1, 2019. We isolated a VDPV1 with 10 nt changes from Sabin type 1 in a sample collected in December 2019 in Aldea Cruz Blanca, SJS, and reported it to the MoH in May 2020. The VDPVs were not genetically linked to any previously sequenced VDPV1 or VDPV3 worldwide. As of July 2021, no other VDPVs have been detected.

### Event Investigation

We identified no cases of AFP from community case searches in which 1,580 children were screened. We isolated no VDPVs from stool samples collected among 61 healthy children sampled (20 children each for the first 2 VDPV detections and 21 children for the third detection).

We conducted a retrospective case review during January 1, 2018–August 31, 2020, in 2 phases after the VDPV notifications in July 2019 and May 2020; we reviewed 3,342,166 records from 42 national hospitals. The reviewers identified 7,318 (0.2%) persons with paralysis diagnoses, of whom 150 (2.1%) met the case definition for AFP. Of those, 56 (37%) cases had not been reported to the AFP surveillance system. No potential cases of primary immunodeficiency from the affected municipalities were found from national hospital-based information systems.

### Routine AFP Surveillance

During 2017–2020, AFP yearly incidence met the target of 1 case/100,000 children in 2019. In 2017, 2018, and 2020, AFP incidence rate was 0.6–0.9 cases/100,000 children. Overall, 164 (76%) of 214 cases reported in 2017–2020 had an adequate sample collected, and 87 (41%) were investigated within 48 hours of being reported (Table 1).

**Table 1:** Surveillance indicators for acute faccid paralysis, Guatemala, 2017–2020*

Year	No. reported AFP cases	Incidence rate per 100,000 children <15 y	Adequate sample collected, no. (%)	Investigation within 48 h, no. (%)
Expected value	55	1.0	≥80%	≥80%
2017	46	0.7	29 (63)	NA
2018	59	0.9	50 (85)	11 (19)
2019	72	1.1	55 (77)	49 (68)
2020	37	0.6	30 (81)	27 (73)

*Information not systematically collected before 2018. Source: Department of Epidemiology, Guatemalan Ministry of Health. NA, not available.

### Vaccination Coverage and OPV/MMR Supplementary Immunization Campaign

During 2017–2020, the percentage of children <1 year of age who received 3 doses of polio vaccine as 2 doses of IPV and 1 dose of bOPV was 84.8%–89.8% at the national level (Table 2). Vaccination coverage for that vaccination series in the health areas where VDPVs were isolated was 62.1%–75.3% during the same period.

**Table 2:** Polio vaccination coverage in children <1 y of age, Guatemala, 2017–2020*

Year	Dose	Villa Nueva			San Juan Sacatepequez			National	
		Population	No. (%) vaccinated		Population	No. (%) vaccinated		Population	No. (%) vaccinated
2017	Polio 3	8,339	6,282 (75.3)		6,159	3,824 (62.1)		381,396	342,437 (89.8)
2018	Polio 3	8,296	6,055 (73.0)		6,141	3,942 (64.2)		382,841	324,761 (84.8)
2019	Polio 3	7,252	5,495 (75.8)		5,856	4,056 (69.3)		366,448	320,963 (87.6)
2020	Polio 3	6,671	5,570 (83.5)		5,513	4,252 (77.1)		340,876	304,898 (89.4)
2019	bOPV	57,979	54,942 (94.8)		41,639	37,969 (91.2)		2,649,334	2,463,881 (93.0)

*The 2019 nationwide bOPV campaign targeted children 6 mo to <7 y of age. Polio 3 indicates a third dose of oral or injectable polio-containing vaccine. bOPV, bivalent oral poliovirus vaccine. Source: National Immunization Program, Guatemala Ministry of Health.

During the national bOPV/MMR vaccination campaign conducted in September–November 2019 in response to the VDPV detections, 93.0% of children 6 months to <7 years of age received 1 dose of bOPV. The campaign coverage reached 94.8% among children 6 months to <7 years of age in VNA and 91.2% in SJS. 

### OBRA Results

The 2021 OBRA conducted in Guatemala found no evidence of circulating VDPVs in Guatemala and that the MoH response planning and coordination after VDPV identification in 2019 environmental surveillance samples was appropriate. The 3 VDPVs detected in wastewater samples were classified as aVDPVs.

## Discussion

In Guatemala, the environmental surveillance of poliovirus in wastewater detected 3 genetically unrelated VDPVs in 1 year. After careful revision of the MoH response and investigation, an international team of experts classified those 3 events as aVDPVs.

Although the Americas region was declared free from polio in 1994, recent events remind us that sustaining this status is a continuous challenge. In June 2022, a VDPV2 was isolated in an unvaccinated person in New York, New York, USA, and classified as cVPDV2 after being isolated from wastewater (*8*). This virus, genetically linked to other VDPV2 viruses identified in Israel and the United Kingdom (*20*), demonstrates the global threat that importation of poliovirus from anywhere in the world represents. In March 2023, a case of VDPV1 was detected in an unvaccinated child from a community with low vaccination coverage in Peru (*21*). The virus presented 31 nt changes compared with the type 1 Sabin virus and was not genetically linked to any VDPV1 identified in the world. This case is an example of the imminent risk for reemergence of poliomyelitis through VDPVs emerging in communities with low OPV vaccination coverage.

The OBRA team concluded that there was enough evidence to consider that none of the 3 VDPVs isolated in Guatemala had given rise to a clinical case of poliomyelitis in the country; however, those findings highlight the need to improve vaccination coverage and AFP surveillance to prevent or timely detect poliovirus reemergence. Through repeated workshops using the OBRA report structure (AFP surveillance, environmental surveillance, immunizations, and community mobilization), the Guatemala MoH translated results and recommendations from the evaluation into a roadmap to mitigate the risk for polio reemergence.

In Guatemala, 1 of 3 AFP cases detected through retrospective hospital-based case search had not been identified through routine surveillance. Such an underperforming surveillance system could place the country at risk of failing to detect clinical poliomyelitis in a timely manner. To improve AFP surveillance, the MoH implemented a daily zero-notification process, in which hospitals daily ascertain the absence of AFP cases in their admitted patients. After the OBRA evaluation, MoH strengthened the national surveillance team through increased staff and interinstitutional (national laboratory, epidemiology department and national immunizations program) training supported by national and international experts. MoH and non-MoH hospitals made efforts to improve AFP notification; AFP notifications increased to 0.9 cases/100,000 children in 2021 and 1.2 cases/100,000 children in 2022. Environmental surveillance continued through monthly wastewater sample collection in the same sampling sites; as of December 2022, no further VDPV or WPV had been detected.

National vaccination coverage with 3 doses of polio vaccine was 85% in 2018, the year before the VDPV isolations; at that time, coverage was 73% in VNA and 64% in SJS. Vaccination coverage in areas with poor access to clean water allowed for VPDVs to emerge and be detected in wastewaters. The first two VDPVs were notified in July 2019, which was 2 months before the launch of a planned national catch-up bOPV/MMR vaccination campaign. Notification of the VDPVs in accordance with international health regulations led to international awareness as well as technical and financial assistance from PAHO, the United Nations Children’s Fund, and GPEI for the MoH to successfully implement its vaccination campaign, reaching 93% of children 6 months to <7 years of age vaccinated with a dose of bOPV. In accordance with GPEI guidelines for polio outbreak response, vaccination campaigns should be organized after VDPV is classified as cVDPV (*22*). After the isolation of the third VDPV and its notification in May 2020, case investigation led to its classification as aVDPV; no further vaccination campaign was organized. Since the OBRA evaluation, declining polio vaccination coverage during the COVID-19 pandemic (*7*,*23*,*24*) has built a pool of susceptible children representing a risk for polio reemergence (*25*,*26*). National vaccination coverage for 3 doses of polio-containing vaccine in Guatemala has declined from 89% in 2020 to 76% in 2022 (27). Routine immunization programs in Guatemala must be intensified through outreach strategies and a better understanding of local issues contributing to low vaccination coverage. Since November 2022, MoH has implemented local workshops to identify reasons for low performance of vaccine preventable disease surveillance and immunization programs. During those 2-day workshops, participants from local and central MoH, municipalities, community leaders, and health service users have jointly developed mitigation plans using a problem tree analysis methodology.

Because of an elevated risk for polio reemergence, adequate response preparedness is essential for timely response to poliovirus detection. The OBRA team concluded that the outbreak response planning and implementation of activities in Guatemala were adequate according to international standards defined by GPEI. Existing, regularly updated guidelines and periodic outbreak preparedness training were key elements that enabled the Guatemala MoH to respond adequately to these events.

In conclusion, the detection of 3 unrelated VDPVs in Guatemala confirmed the strong risk for poliomyelitis emergence in sites with low vaccination coverage. Although no evidence of a circulating VDPV was found, improving polio vaccination coverage is critical to prevent new VDPV emergences and spread. Strengthening AFP surveillance for timely detection of clinical cases is essential for a rapid response to be implemented in all areas of the country. In high-risk countries, complementing AFP surveillance with risk-assessed environmental surveillance is of great value for early detection of reverted vaccine virus before they generate poliomyelitis cases or can circulate widely in the community.

Appendix Additional information on response to vaccine-derived polioviruses detected through environmental surveillance, Guatemala, 2019.
