# Supplementary material for: Response to Vaccine-Derived Polioviruses Detected through Environmental Surveillance, Guatemala, 2019
Source: Emerg Infect Dis. 2023 Aug;29(8):1524–30. doi: 10.3201/eid2908.230236 (PMC10370855; doi:10.3201/eid2908.230236)
Supplement: Appendix — Additional information on response to vaccine-derived polioviruses detected through environmental surveillance, Guatemala, 2019. [file 23-0236-Techapp-s1.pdf]

Article DOI: http://doi.org/10.3201/eid2908.230236

Response to Vaccine-Derived Polioviruses Detected through Environmental Surveillance, Guatemala, 2019

Appendix

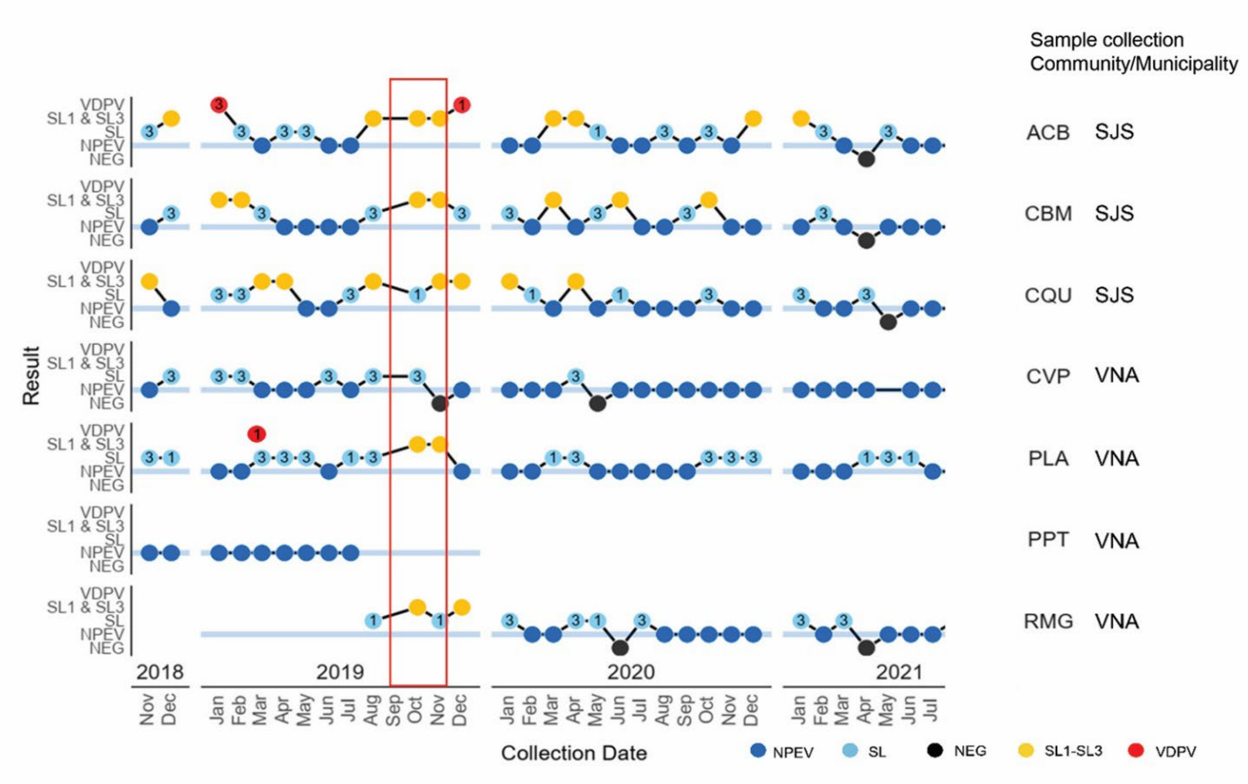

Appendix Figure. Environmental surveillance results in locations in Guatemala, 2018–2021. Red rectangle indicates the timing of the Polio/MMR national vaccination campaign. ACB, Aldea Cruz Blanca; CBM, Bodega Municipal; CQU, Ciudad Quetzal; CVP, Colinas de Villa Nueva; NEG, negative result, no virus detection; NPEV, non-polio enterovirus; PLA, Rio Platanitos; PPT, Ciudad Peronia/Planta de Tratamiento de Aguas; RMG, Rio Magdalena; SJS, San Juan Sacatepéquez; SL, Sabin-like virus; VDPV, vaccine-derived poliovirus; VNA, Villa Nueva.
